# Deep learning-based prediction of HER2 status from breast diffusion-weighted MRI

**DOI:** 10.3389/fonc.2026.1863720

**Published:** 2026-10-05

**Authors:** Yao Zhang, Xinran Kan, Jiaqi Wang, Jie Peng, Jun Cai, Yajun Ma, Cheng Wang

**Affiliations:** 1 Department of Radiology, The First Affiliated Hospital of Yangtze University, Jingzhou, China; 2 School of Physics and Optoelectronic Engineering, Yangtze University, Jingzhou, China; 3 Department of Oncology, The First Affiliated Hospital of Yangtze University, Jingzhou, China; 4 Department of Radiology, University of California, San Diego, San Diego, CA, United States

**Keywords:** breast cancer, classification, deep learning, human epidermal growth factor receptor 2, magnetic resonance imaging

## Abstract

**Background and objective:**

The accurate classification of human epidermal growth factor receptor 2 (HER2) status is very important for the diagnosis and treatment of breast cancer. However, the biopsy, the current gold standard for differentiating HER2 status, is invasive and time-consuming. To overcome these drawbacks, a novel deep learning model was developed to differentiate HER2-negative and HER2-positive status in breast cancer solely based on diffusion-weighted imaging (DWI).

**Materials and methods:**

This retrospective study included 239 women patients confirmed with breast cancer from two local medical centers. A hybrid CNN-Transformer DL model was proposed, which took DWI images (the ADC maps, DWI images with b = 0 s/mm² and b = 800 s/mm²) as inputs and output the classification of HER2-negative and HER2-positive status. Classification by the proposed DL model was quantitatively compared to the classification by the other benchmark DL models and two clinical experts.

**Results:**

Data of the 239 patients (mean age, 49.4 ± 10.0 years) were separated into a training set (n = 156), an internal test set (n = 39), and an external test set (n = 44). On the internal test set, the proposed DL model performed numerically better than the best benchmark DL model (area under the curve [AUC]: 0.93 vs. 0.89; accuracy: 0.90 vs. 0.85). On the external test set, the proposed model also performed numerically better than the best benchmark model (AUC: 0.91 vs. 0.87; accuracy: 0.84 vs. 0.82), and significantly better than the two clinical experts (AUC: 0.91 vs. 0.65 vs. 0.63; accuracy: 0.84 vs. 0.61 vs. 0.57).

**Conclusion:**

This study demonstrates the promise of combining DWI and DL for the classification of HER2 status in breast cancer, and it may potentially serve as a non-invasive adjunct or decision-support tool.

## Introduction

Breast cancer is a common and aggressive malignant tumor that poses a significant threat to women’s health globally (1). In the diagnosis and management of breast cancer, human epidermal growth factor receptor 2 (HER2) status is a critical biomarker (2). Compared with HER2-negative patients, HER2-positive patients can benefit from the targeted therapy trastuzumab (3). Therefore, accurate assessment of HER2 status is key to developing effective treatment plans in breast cancer therapy. Currently, according to clinical guidelines (4), the invasive biopsy is the gold standard for diagnosing HER2 status. Immunohistochemistry (IHC) and fluorescence *in situ* hybridization (FISH) are standard biopsy-based assays. HER2-positive status is defined as an IHC score of 3+ or an IHC score of 2+ with FISH amplification. Conversely, HER2-negative status is defined as an IHC score of 0 or 1+, or an IHC score of 2+ without FISH amplification. Nevertheless, this approach is invasive and time-consuming (5). Therefore, there is an urgent need for an accurate, rapid, and non-invasive method to determine HER2 status in breast cancer diagnosis and therapy.

Currently, magnetic resonance imaging (MRI) has shown potential in breast cancer diagnosis by providing comprehensive visualization and characterization of the tumor (6). In previous studies (7–9), radiomics has been applied to distinguish HER2 statuses in breast MRI. Peng et al. utilized clinical radiomics nomograms to classify HER2 status from the T2-weighted imaging (T2WI), the b-value images and apparent diffusion coefficient (ADC) maps of diffusion-weighted imaging (DWI), and delayed contrast-enhanced T1-weighted imaging, achieving an area under the curve (AUC) of 0.77-0.80 (10). Ramtohul et al. utilized the least absolute shrinkage and selection operator (LASSO) with histological integration to classify HER2 status from T2WI and dynamic contrast-enhanced T1-weighted imaging (DCE-T1WI), achieving an AUC of 0.77-0.80 (11). Zheng et al. utilized LASSO regression to classify HER2 status from T2WI with fat suppression, the b-value images and ADC maps of DWI, and DCE-T1WI, achieving an AUC of 0.72-0.81 (12). These studies confirm the feasibility of MRI radiomics for discriminating HER2 status. Nevertheless, radiomics requires extensive, time-consuming manual feature extraction and demonstrates moderate accuracy, posing challenges for clinical implementation.

Deep learning (DL) has shown significant success in medical image classification by enabling automatic feature extraction (13, 14). Preliminary studies have applied DL in DCE-MRI for HER2 status classification. Liu et al. utilized a Mask-Guided convolutional neural network (CNN) model to distinguish HER2 status breast cancer from DCE-MRI, achieving an AUC of 0.79-0.81 (15). Dai et al. utilized a CNN-based DL model to predict HER2 status from DCE-T1WI, achieving an AUC of 0.75-0.82 (16). Zhang et al. utilized a Vision Transformer (ViT) model to predict HER2 status from DCE-T1WI, achieving an AUC of 0.71-0.86 (17). Zhang et al. utilized a CNN-based model, integrating mammography, ultrasound, and DCE-MRI to assess HER2 status, achieving an AUC of 0.83-0.87 (18). Fan et al. utilized CNN models including ConvNeXt V2 and 3D ResNet, and a combined model to predict HER2 status from ultrasound and DCE-MRI, achieving an AUC of 0.80-0.90 (19). These studies demonstrate the feasibility of using DL with MRI to predict HER2 status in breast cancer. Nevertheless, these methods all rely on DCE-MRI, while DCE-MRI needs invasive contrast agent administration and poses patient-related risks, such as allergic reactions or potential organ toxicity (20). Additionally, the current DL models are relatively simple, and the prediction accuracy is limited. Using more sophisticated DL models is anticipated to improve the accuracy of HER2 classification.

Based on the above, we intended to develop a hybrid CNN-Transformer model to accurately distinguish HER2-negative and HER2-positive status from non-invasive breast DWI images. Physiologically, the increased vascular permeability and extracellular fluid associated with HER2-positive tumors result in less restricted diffusion, manifesting as elevated ADC values on DWI (21). These establish a direct link between DWI and the underlying biology of HER2-positive disease. Thus, the ADC maps alongside DWI images at b = 0 and b = 800 s/mm² were employed as the inputs to the DL model. Furthermore, to fully leverage the latent features embedded in DWI data for enhanced classification, we proposed a sophisticated hybrid CNN-Transformer model, which can effectively capture both local and global image features to achieve a high classification accuracy in distinguishing HER2 status. We anticipated that the proposed method could provide valuable support for the personalized diagnosis and treatment of breast cancer.

## Materials and methods

### Study population

From January 1, 2021, to December 31, 2024, the breast cancer patients were retrospectively enrolled from two local medical centers. The exclusion criteria of the patients were as follows: 1) incomplete pathological information of HER2 status; 2) poor DWI image quality; 3) any prior treatment before the MRI scan, including surgery, chemotherapy, or radiotherapy. The local ethical review board approved this retrospective study and waived the requirement to obtain individual informed consent. Finally, a total of 239 patients were included in this study. Among them, 156 and 39 patients (80% vs 20%) from the same center were used as the training set and the internal test set, and 44 patients from another center were used as the external test set. The patient enrollment flowchart was shown in **Figure 1**.

**Figure 1:**
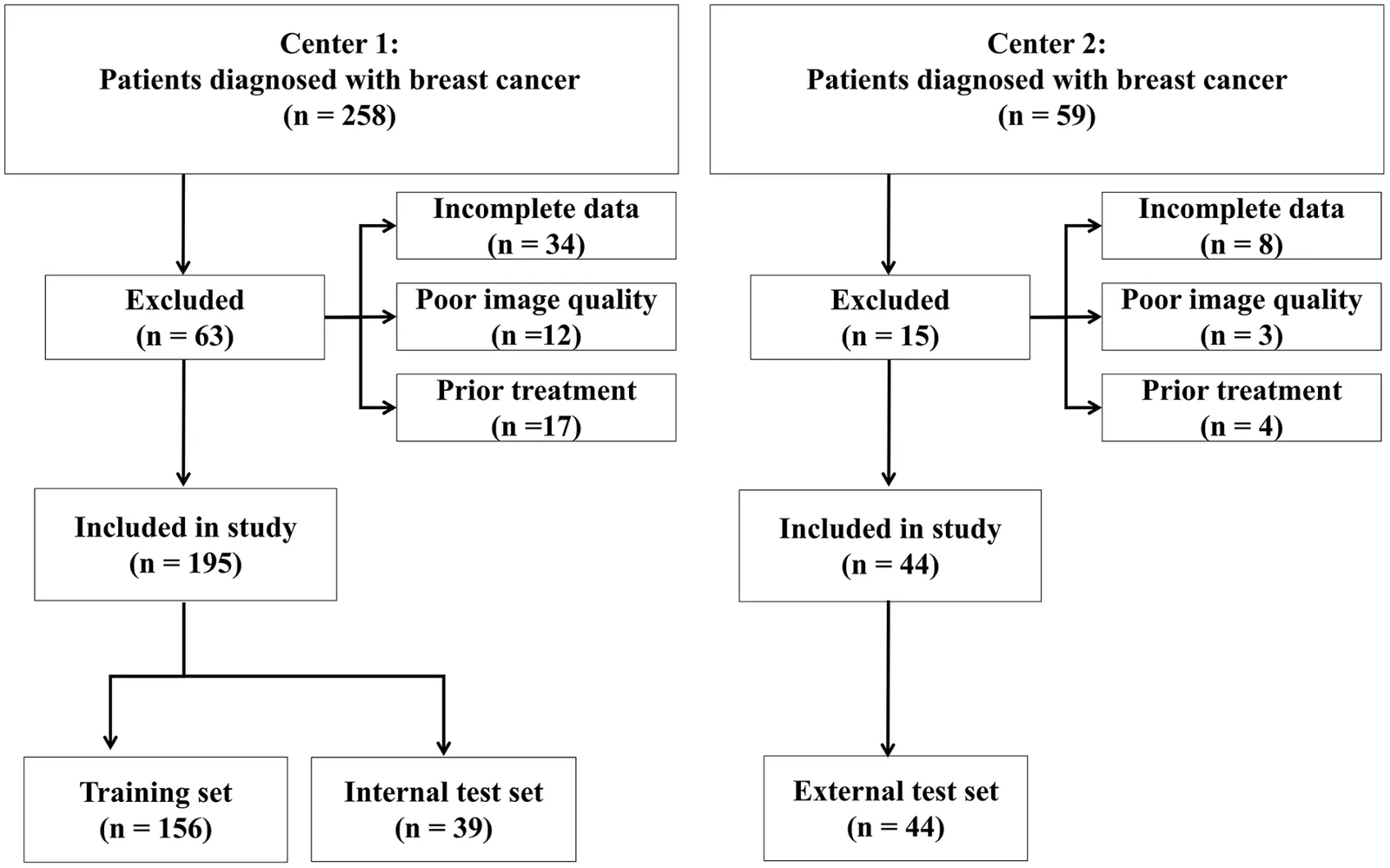
The patient enrollment flowchart.

### Image acquisition and preprocessing

All DWI scans were performed on 1.5 T MRI scanners (Siemens Healthcare) with breast coils. The DWI acquisition parameters were as follows: repetition time (TR) = 5200.0 ms, echo time (TE) = 78.0 ms, field of view (FOV) = 340 × 340 mm², slice thickness = 4.0 mm, slice gap = 1.0 mm, number of slices = 30, and b-values = 0 and 800 s/mm². The ADC map was calculated in a pixel-by-pixel manner from DWI via the manufacturer’s Syngo software.

To reduce the requirements of computer hardware and decrease the training time of the DL model, all ADC maps and DWI images were resized to 128 × 128 × 32 pixels. The grayscale values of all the images were also normalized to the range (0, 1). Besides, online data augmentation was applied during training: in each epoch, each training sample was augmented with a probability of 0.5, with random in-plane rotations within ±10° and random scaling by a factor ranging from 0.90 to 1.10 (22). Augmentation was performed after patient-level data partitioning and was restricted to the training set; all images from the same patient were assigned to the same dataset, and no image from any internal or external test-set patient was used in model training or augmentation, thus precluding data leakage.

The IHC and FISH results for each patient were obtained from surgical specimens. Based on these results and in accordance with the American Society of Clinical Oncology/College of American Pathologists guidelines (4), two pathologists classified the samples as HER2-positive or HER2-negative.

### The hybrid CNN-Transformer model

The workflow of the proposed hybrid CNN-Transformer model was presented in **Figure 2**. The model took DWI images (the ADC maps, DWI images with b = 0 s/mm², and DWI images with b = 800 s/mm²) as inputs and output the classification of HER2-negative and HER2-positive status. No manual tumor segmentation or ROI cropping was performed; the full images were used as model inputs, a strategy previously used in breast MRI deep-learning classification studies (23). The architecture of the model was modified from our prior work (24, 25), which was composed of three main modules: the adaptive hybrid pooling (AHP) module, the feature fusion CNN (FF_CNN) module, and the 3D channel attention transformer (3DCA_Trans) module. The AHP module maximized information retention during down-sampling through the adaptive aggregation of multiple pooling strategies (max pooling and average pooling). The FF_CNN module, which concatenates the shallow-layer and deep-layer features, was designed to preserve information and mitigate gradient-related issues in deep networks. Inspired by the DenseNet (26) architecture, we deepened our network by sequentially repeating the composite block of AHP module and FF_CNN module four times, where the number of FF_CNN module units appended after the AHP module followed an increasing sequence (i.e., 6, 12, 24, and 16 for the first, second, third, and fourth block, respectively). The 3DCA_Trans module employed a channel attention mechanism to extract the global features from the 4D feature map, ultimately outputting the classification result. The more detailed and comprehensive descriptions of the proposed hybrid CNN-Transformer model were additionally provided in the **Supplementary Material** (**Supplementary Figure 1**; **Supplementary Table 1**).

**Figure 2:**
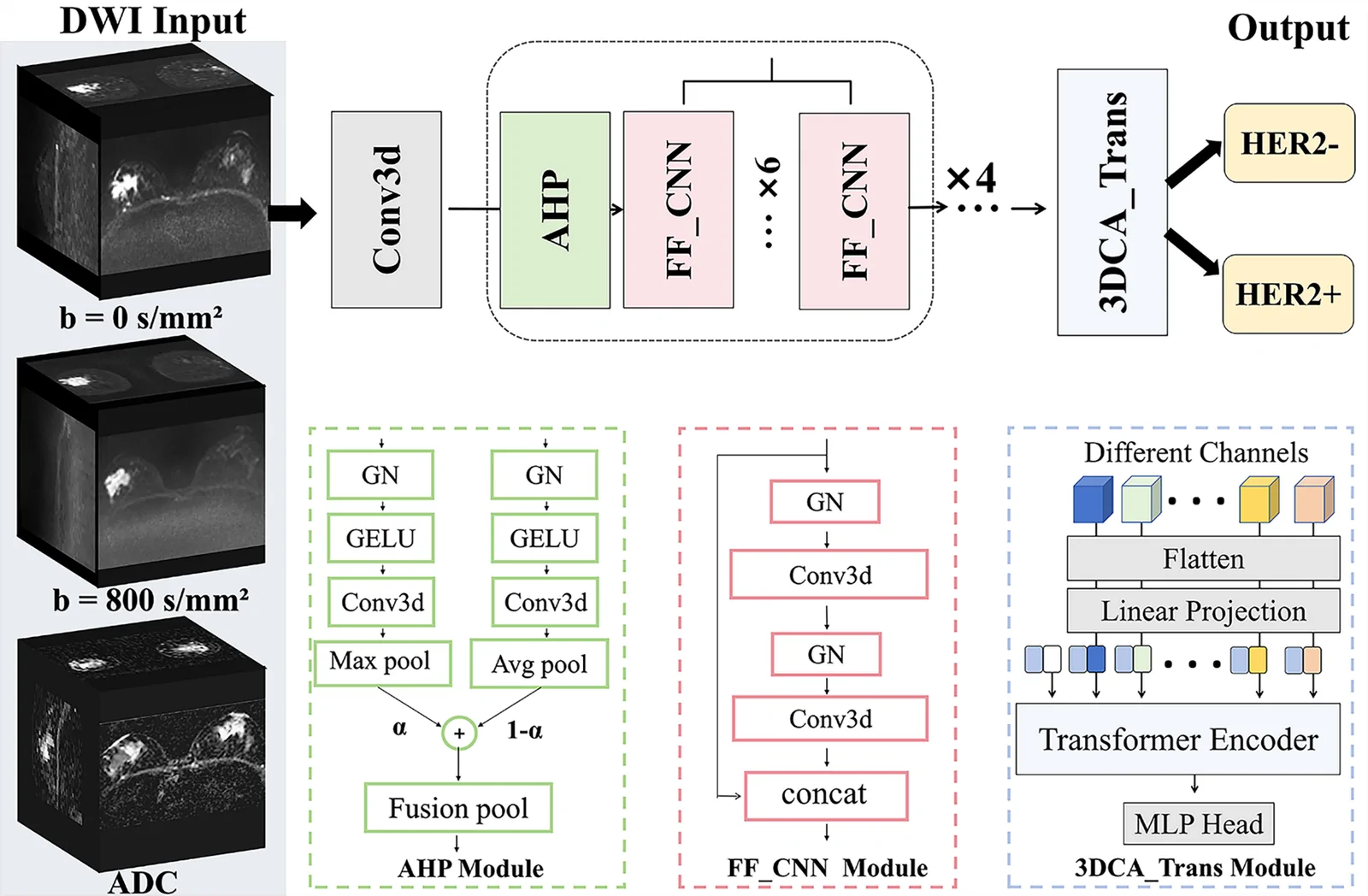
The workflow of the proposed hybrid CNN-Transformer model for the HER2 classification in breast cancer, which took only the DWI images (ADC maps, DWI images with b = 0 s/mm², and DWI images with b = 800 s/mm²) as inputs and output the classification of HER2-negative and HER2-positive status.

The Grad-CAM heatmaps (16) were generated from the feature maps of the last convolutional block of the CNN feature extractor (the final spatial feature maps before the Transformer module). Gradients of the HER2-positive output logit were backpropagated to these feature maps and global-average-pooled to derive channel-wise weights, which were combined to produce heatmaps overlaid on the DWI images. The color gradient from blue through green and yellow to red indicates progressively increasing model attention. All heatmaps were visually reviewed by the two breast radiologists to confirm that the highlighted regions corresponded to the tumor area.

The loss function of the model was set to the cross-entropy with the Adam optimizer. During the training, the batch size was set as 2 owing to GPU memory constraints, the epoch was fixed at 100, the learning rate was initialized at 0.00005 followed by a cosine learning rate scheduler, and the weight decay was 0.00001. The learning rate was adjusted only according to the convergence behavior of the training-set loss curve. No early stopping was applied, and the network parameters at the final epoch were used for all evaluations. Neither the internal nor the external test set was involved in any stage of model development, including hyperparameter tuning, checkpoint selection, and threshold selection. No separate validation set was used, as no development decision depended on evaluation data. The training loss curve is provided in the **Supplementary Material** (**Supplementary Figure 3**). In this work, the model was implemented on PyTorch 2.3 with an NVIDIA RTX 4060 GPU (8 GB memory), and the training time was 3 minutes per epoch.

### The benchmark DL models

In this subsection, we presented several comparison models commonly used in the previous DL studies (17, 19) for the HER2 status classification. 1) ResNet (27), which utilizes the residual connections to support the training of deep networks and extract hierarchical image features for classification. 2) DenseNet (26): which adopts dense connectivity between layers to enable feature reuse across different network layers. 3) ConvNeXt V2 (28): which utilizes a modernized convolutional structure to capture multi-scale image features for image classification. 4) ViT (29): which applies the Transformer architecture directly to image classification by splitting images into patches and treating them as 2D token sequences. To ensure a fair comparison, all benchmark models were trained and evaluated under the same preprocessing, augmentation, and optimization settings.

### Reader evaluation

A reader study was designed to compare the classification performance between the proposed hybrid CNN-Transformer model and the clinical experts. Two experienced breast radiologists (Y.Z. and J.P.) from the local hospital participated, who both had over 10 years of clinical experience. The DWI images in the external test set were presented to the readers in random order. For each case, each reader independently assigned a confidence score ranging from 0 to 100 in increments of 5, indicating the likelihood of HER2-positive status. Reader ROC curves were constructed from these confidence scores. For binary classification, a pre-specified threshold of 50 was applied, with scores < 50 considered HER2-negative and scores ≥ 50 considered HER2-positive. Both readers were blinded to the histopathological results, the model outputs, and each other’s scores.

### Statistical analysis

In this work, the quantitative metrics of accuracy (ACC), sensitivity (SEN), specificity (SPE), F1 score, and AUC under the receiver operating characteristic (ROC) curve were used to evaluate the classification performance. A pre-specified fixed threshold of 0.5 was uniformly applied to the outputs of all models in all datasets to derive binary classifications. The patient characteristics between different groups were analyzed using the ANOVA test for continuous variables and the Pearson’s chi-square test for categorical variables. The DeLong test was performed to verify the diagnostic superiority of findings. For ACC, SEN and SPE, paired-difference and confidence intervals were calculated using Newcombe method 10, and *P* values were obtained using the McNemar test. For F1 score, paired-difference and confidence intervals were estimated using 1000 paired row-bootstrap resamples, and two-sided *P* values were calculated using the null-centered paired-bootstrap test. The intraclass correlation coefficient (ICC) of the confidence scores between the two readers was evaluated using a two-way random-effects, absolute-agreement, single-measurement model, i.e., ICC (2,1). *P* values from the pairwise comparisons were considered nominal and exploratory, and no adjustment for multiple comparisons was applied; accordingly, statistical interpretation was based on effect sizes, confidence intervals, and the consistency of differences across cohorts rather than on isolated *P* values. *P* < 0.05 represents the statistical significance. The statistical analysis was performed utilizing R software (version 4.5, The R Foundation, Vienna, Austria). In addition, a model-specific threshold was also determined by maximizing the Youden index on the ROC curve (16); the corresponding results based on the Youden-index threshold are provided in the **Supplementary Material** (**Supplementary Tables 5**–**9**; **Supplementary Figure 2**).

## Results

### Clinicopathological data

**Table 1** showed the clinical characteristics of the included 239 patients. The mean age of the patients was 49.4 ± 10.0 years old. Among the patients, 113 (47.3%) were HER2-positive, and 126 (52.7%) were HER2-negative. There were no significant group differences between the training set, the internal test set, and the external test set in age (*P* = 0.355), HER2 status (*P* = 0.921), histology type (*P* = 0.131), ER status (*P* = 0.543), PR status *(P* = 0.275), and Ki-67 (*P* = 0.051).

**Table 1:** The clinicopathologic characteristics of the included patients.

Characteristic	Training set (N = 156)	Internal test set (N = 39)	External test set (N = 44)	*P* value
Age, years	49.7 ± 9.8	47.3 ± 11.2	50.1 ± 9.5	0.355
HER2 status				0.921
Positive	73 (47)	18 (46)	22 (50)	
Negative	83 (53)	21 (54)	22 (50)	
Histology type				0.131
Invasive cancer	148 (95)	35 (90)	38 (86)	
Other	8 (5)	4 (10)	6 (14)	
ER status				0.543
Positive	87 (56)	23 (59)	21 (48)	
Negative	69 (44)	16 (41)	23 (52)	
PR status				0.275
Positive	67 (43)	16 (41)	13 (30)	
Negative	89 (57)	23 (59)	31 (70)	
Ki-67 (%)				0.051
≥14	115 (74)	27 (69)	24 (55)	
<14	41 (26)	12 (31)	20 (45)	

Data are presented as mean ± standard deviation for continuous variables (Age) and as number (percentage) for categorical variables. HER2, human epidermal growth factor receptor 2; ER, estrogen receptor; PR, progesterone receptor; Ki-67, proliferation index.

### Deep learning classification results

**Table 2** demonstrated the quantitative evaluation results of the proposed hybrid CNN-Transformer model and other benchmark DL models for HER2 status classification in the internal test set (39 patients). The results showed our model achieved the highest performance in AUC, ACC, SPE, and F1 score, and the second highest in SEN. The AUC of the proposed model was 0.93 (95% confidence interval [CI]: 0.82-0.99), and the ACC was 0.90 (95% CI: 0.80-0.97). The SEN of the proposed model was 0.83 (95% CI: 0.65-1.00), the SPE was 0.95 (95% CI: 0.84-1.00), and the F1 score was 0.88 (95% CI: 0.75-0.97). The detailed results regarding paired differences in AUC and other metrics with 95% confidence intervals in the internal test set are provided in the **Supplementary Material** (**Supplementary Table 2**).

**Table 2:** The comparison between the proposed hybrid CNN-transformer DL model and other benchmark DL models for HER2-positive and HER2-negative classification in the internal test set.

Method	AUC	Accuracy	Sensitivity	Specificity	F1 score
ViT	0.82 (0.69, 0.92)	0.79 (0.67, 0.90)	**0.89 (0.73, 1.00)**	0.71 (0.52, 0.89)	0.80 (0.65, 0.92)
DenseNet	0.83 (0.68, 0.95)	0.77 (0.64, 0.90)	0.78 (0.57, 0.95)	0.76 (0.58, 0.93)	0.76 (0.58, 0.89)
ResNet	0.85 (0.72, 0.96)	0.85 (0.72, 0.92)	0.83 (0.64, 1.00)	0.86 (0.70, 1.00)	0.83 (0.67, 0.95)
ConvNeXt	0.89 (0.76, 0.98)	0.85 (0.72, 0.95)	**0.89 (0.73, 1.00)**	0.81 (0.63, 0.95)	0.84 (0.69, 0.95)
**Our Model**	**0.93 (0.82, 0.99)**	**0.90 (0.80, 0.97)**	0.83 (0.65, 1.00)	**0.95 (0.84, 1.00)**	**0.88 (0.75, 0.97)**

The best results are shown in bold. The values in parentheses are confidence intervals. AUC, area under the receiver operating characteristic curve.

**Table 3** demonstrated the quantitative evaluation results of the proposed hybrid CNN-Transformer model and other benchmark DL models for HER2 status classification in the external test set (44 patients). The results showed our model achieved the highest performance in AUC, ACC, SEN, and F1 score. The AUC of the proposed model was 0.91 (95% CI: 0.79-0.99), and the ACC was 0.84 (95% CI: 0.73-0.93). The SEN of the proposed model was 0.91 (95% CI: 0.79-1.00), the SPE was 0.77 (95% CI: 0.58-0.95), and the F1 score was 0.85 (95% CI: 0.73-0.95). The detailed results regarding paired differences in AUC and other metrics with 95% confidence intervals in the external test set are provided in the **Supplementary Material** (**Supplementary Table 3**).

**Table 3:** The comparison between the proposed hybrid CNN-transformer DL model and other benchmark DL models for HER2-positive and HER2-negative classification in the external test set.

Method	AUC	Accuracy	Sensitivity	Specificity	F1 score
ViT	0.81 (0.67, 0.94)	0.82 (0.70, 0.93)	0.86 (0.70, 1.00)	0.77 (0.59, 0.95)	0.83 (0.67, 0.92)
DenseNet	0.82 (0.68, 0.94)	0.66 (0.52, 0.80)	0.77 (0.60, 0.95)	0.55 (0.33, 0.75)	0.69 (0.51, 0.84)
ResNet	0.84 (0.68, 0.96)	0.68 (0.55, 0.82)	0.41 (0.21, 0.61)	**0.95 (0.85, 1.00)**	0.56 (0.33, 0.74)
ConvNeXt	0.87 (0.73, 0.97)	0.80 (0.68, 0.91)	0.77 (0.59, 0.95)	0.82 (0.65, 0.96)	0.79 (0.63, 0.91)
**Our Model**	**0.91 (0.79, 0.99)**	**0.84 (0.73, 0.93)**	**0.91 (0.79, 1.00)**	0.77 (0.58, 0.95)	**0.85 (0.73, 0.95)**

The best results are shown in bold. The values in parentheses are confidence intervals. AUC, area under the receiver operating characteristic curve.

**Figure 3** displayed the classification ROC curve results of the proposed hybrid CNN-Transformer model and other benchmark DL models. **Figure 3A** demonstrated that our model achieved the highest AUC among all models in the internal test set. **Figure 3B** also demonstrated that our model achieved the highest AUC among all models in the external test set.

**Figure 3:**
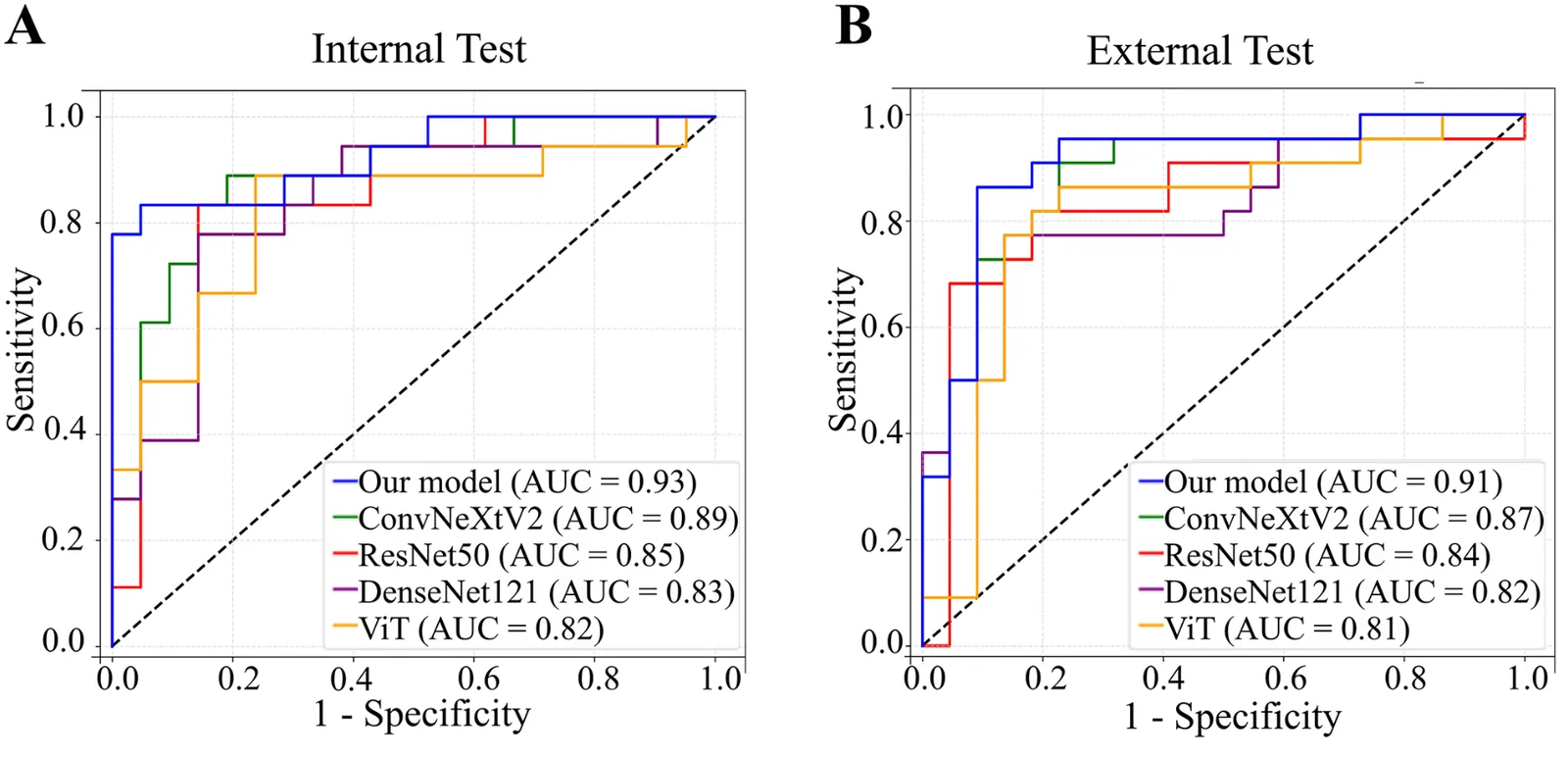
The comparison between the receiver operating characteristic (ROC) curves of different deep learning models in **(A)** the internal test set and **(B)** the external test set.

**Figure 4** showed the confusion matrix of the classification results by the proposed hybrid CNN-Transformer model. In the internal test set (n = 39), the proposed model correctly identified 15 HER2-positive and 20 HER2-negative samples, with only 1 false positive and 3 false negatives, respectively. In the external test set (n = 44), the proposed model correctly identified 20 HER2-positive and 17 HER2-negative samples, with only 5 false positives and 2 false negatives, respectively.

**Figure 4:**
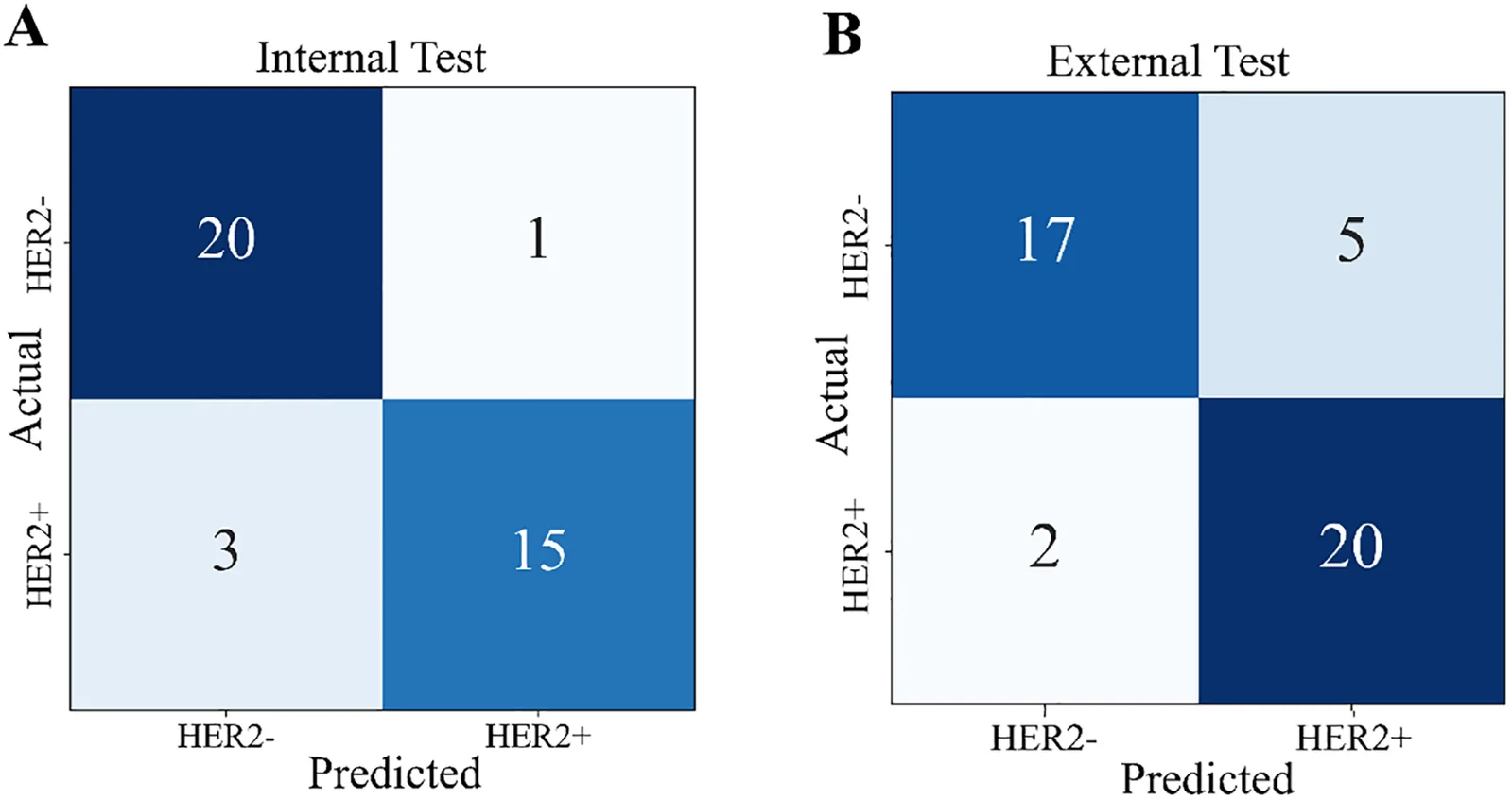
The confusion matrices for HER2 status classification by the proposed hybrid CNN-transformer model in **(A)** the internal test set and **(B)** the external test set.

**Figure 5** demonstrated the heat map results of the proposed hybrid CNN-Transformer model for 3 patients. Columns 1 to 4 of the figure displayed the patient’s DWI images (b = 800 s/mm²), ADC maps, heatmaps generated by the proposed DL model, and biopsy pathology images, respectively. The color gradient from blue to green, then to yellow, and finally to red in the heatmaps indicated a progressive increase in the model’s attention. It could be seen that the model’s focus (red regions of heat maps) coincided with the pathological area highlighted in the DWI images.

**Figure 5:**
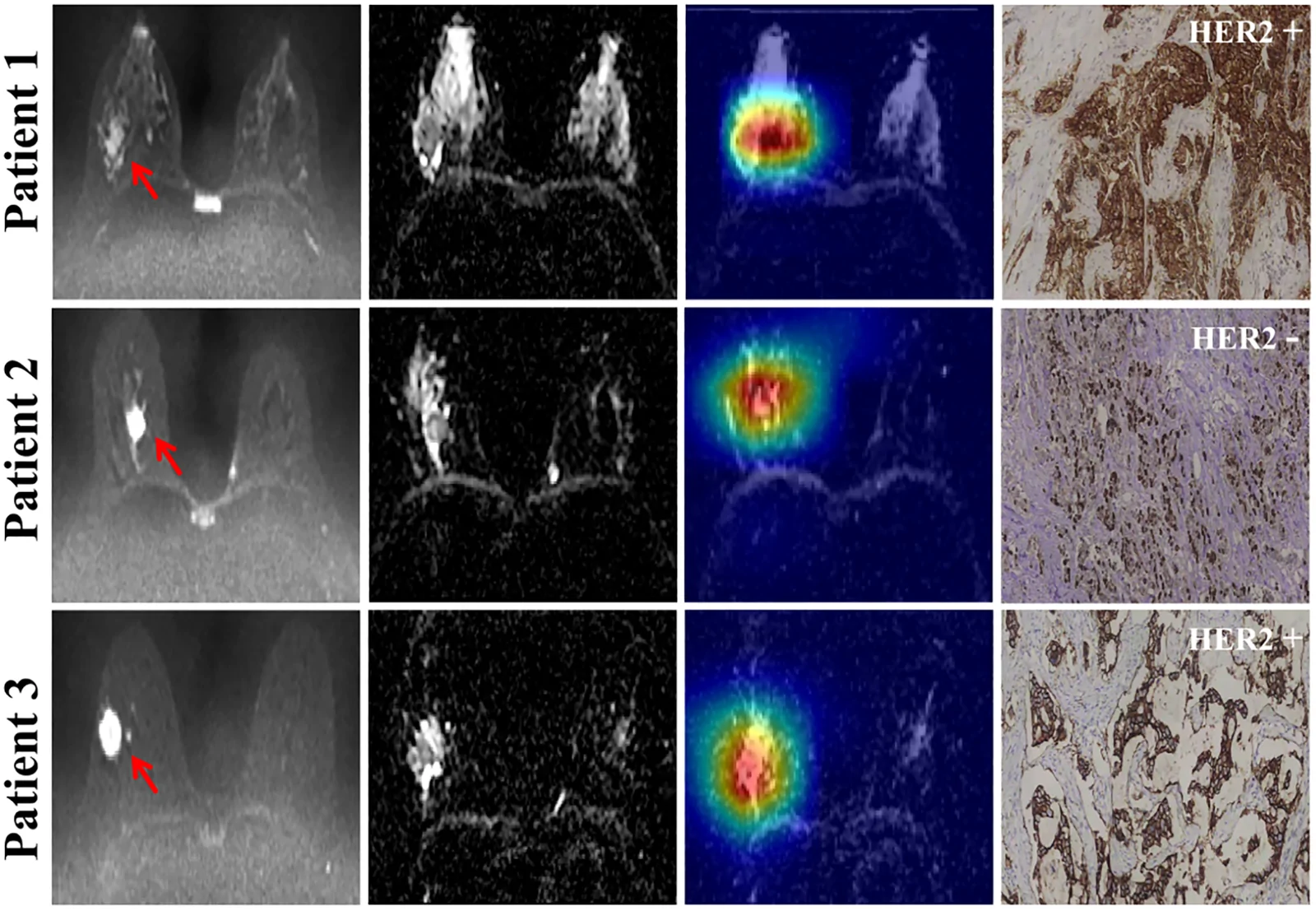
The visualization of the inputs, heatmaps, and labels of the proposed DL model with three patient examples. The columns 1–4 represent: DWI images with b = 800 s/mm², ADC map, heatmaps, and HER2 pathological examination maps. Patient 1 was a woman with HER2-positive breast cancer. Patient 2 was a woman with HER2-negative breast cancer. Patient 3 was a woman with HER2-positive breast cancer. The red arrows in the DWI images indicate the lesions. The color gradient from blue to green, then to yellow, and finally to red in the heatmaps indicates a progressive increase in the DL model’s attention.

### Comparison between the DL model and clinical experts

**Table 4** demonstrated the quantitative evaluation results of the clinical experts (Reader 1 and Reader 2) for HER2 status classification in the external test set (44 patients). The results showed that the proposed hybrid CNN-Transformer model achieved significantly higher AUC, ACC, and F1 score than both clinical experts, together with numerically higher SEN and SPE.

**Table 4:** The comparison between the proposed DL model and two clinical experts (reader 1 and reader 2) for HER2-positive and HER2-negative classification in the external test set.

Method	AUC	Accuracy	Sensitivity	Specificity	F1 score
Reader 1	0.63 (0.44, 0.77)	0.57 (0.41, 0.70)	0.64 (0.41, 0.83)	0.50 (0.29, 0.70)	0.60 (0.40, 0.73)
Reader 2	0.65 (0.47, 0.80)	0.61 (0.48, 0.75)	0.59 (0.38, 0.79)	0.64 (0.42, 0.82)	0.61 (0.42, 0.76)
**Our Model**	**0.91 (0.79, 0.99)**	**0.84 (0.73, 0.93)**	**0.91 (0.79, 1.00)**	**0.77 (0.58, 0.95)**	**0.85 (0.73, 0.95)**

The best results are shown in bold. The values in parentheses are confidence intervals. AUC, area under the receiver operating characteristic curve.

The AUC of the Reader 1 was 0.63 (95% CI: 0.44-0.77), and the ACC was 0.57 (95% CI: 0.41-0.70). The AUC of the Reader 2 was 0.65 (95% CI: 0.47-0.80), and the ACC was 0.61 (95% CI: 0.48-0.75). The DeLong test demonstrated a significant difference between our model and Reader 1 (*P* = 0.004). The DeLong test demonstrated a significant difference between our model and Reader 2 (*P* = 0.009). The intraclass correlation coefficient (ICC) for the ratings by the two clinical experts was 0.83 (95% CI: 0.72-0.91), indicating good agreement. The detailed results regarding paired differences in AUC and other metrics with 95% confidence intervals between DL model and clinical experts are provided in the **Supplementary Material** (**Supplementary Tables 10**, **11**).

**Figure 6** displayed the ROC and the confusion matrix results of the two clinical experts. **Figure 6A** demonstrated that the AUC under the ROC of the proposed DL model outperformed the clinical experts. **Figure 6B** showed Reader 1 correctly identified 14 HER2-positive and 11 HER2-negative samples, with 11 false positives and 8 false negatives, respectively. **Figure 6C** showed Reader 2 correctly identified 13 HER2-positive and 14 HER2-negative samples, with 8 false positives and 9 false negatives, respectively.

**Figure 6:**
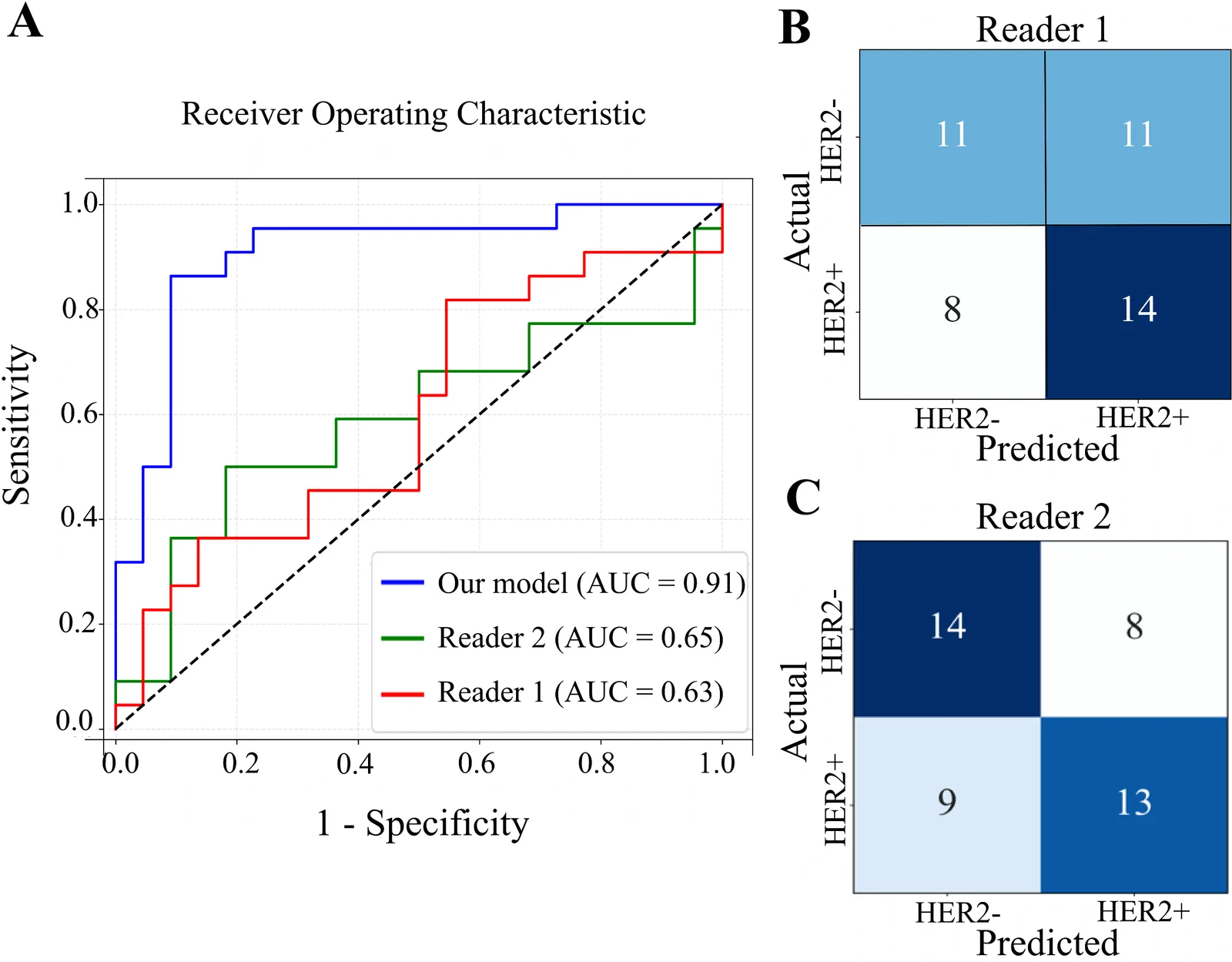
The comparison between the clinical experts and the proposed DL model. **(A)** The ROC curves of two experts (Reader 1 and Reader 2) and the DL model; **(B)** The confusion matrix of the Reader 1; **(C)** The confusion matrix of the Reader 2.

## Discussion

The accurate classification of positive and negative HER2 status is very important for the diagnosis and treatment of breast cancer. Compared with the conventional invasive biopsy, MRI presents a promising classification way with the advantages of being less time-consuming and non-invasive (30). Previous studies have used DCE-MRI images and DL to predict the HER2 status, showing a potential performance (15–20). Nevertheless, DCE-MRI requires the injection of the contrast agent and introduces new physical health risks. To solve those challenges, we first used the only non-invasive DWI sequences and DL to predict HER2 status, and proposed a sophisticated hybrid CNN-Transformer model to achieve this classification. Our study further demonstrates the feasibility of non-invasive HER2 classification using DWI, and the proposed method shows significant promise as a clinical tool for breast cancer management. In this way, our work could help provide an accurate, rapid, and non-invasive method for the classification of HER2 status.

The results showed our model achieved a high performance with an AUC of 0.93 in the cohort for HER2 status classification. This is mainly attributed to the proposed hybrid CNN-Transformer model, which was specifically designed for breast DWI. While numerous studies have demonstrated the superiority of the Transformer architecture in medical image classification, this often depends on the availability of large datasets (31, 32). This is consistent with our experimental findings, where the pure Transformer architecture (ViT model) exhibited only moderate performance (AUC = 0.82) on our cohort. To improve the accuracy, our model is designed to integrate both the local and global features of DWI images: the CNN extracts tumor features, while the proposed 3D Transformer module (3DCA_Trans) captures the spatial relationships with the surrounding breast tissue from 4D image features. Additionally, two custom modules (AHP and FF_CNN) are engineered to preserve feature integrity throughout down-sampling, and as network depth increases, together further strengthening this hybrid model. Comprehensive comparisons showed that the proposed model achieved consistently numerically higher AUC and accuracy than all benchmark models in both test sets (paired AUC differences: 0.04–0.11), although most differences did not reach statistical significance; several were significant, including sensitivity (*P* = 0.002) and F1 score (*P* = 0.021) versus ResNet and accuracy versus DenseNet (*P* = 0.033) in the external test set (**Supplementary Tables 2**, **3**). The limited sample size of the test cohorts may partly account for these largely non-significant results; consistent with this possibility, the number of significant comparisons increased with sample size, from 1 of 20 in the internal test set (n = 39) to 3 of 20 in the external test set (n = 44) and 6 of 20 in the pooled cohort (n = 83) (**Supplementary Table 4**). In addition, the already strong performance of the benchmark models leaves limited room for large absolute improvements, and with only 18–22 positive or negative cases in each test set, sensitivity and specificity vary in discrete steps of approximately 4–6 percentage points per patient, both of which inherently limit the magnitude and resolution of pairwise differences. Together with the consistent direction of all paired differences, these findings suggest a modest advantage of the proposed model that warrants confirmation in larger cohorts.

On the other hand, the strong performance of our model in predicting from DWI is attributed not only to its DL architecture but also to the intrinsic physiological link between DWI signals and HER2 status. DWI is associated with HER2 status through the pathophysiological mechanism: HER2 signaling drives angiogenesis and increases vascular permeability. This process increases extracellular fluid volume, reduces water diffusion restriction, and increases the ADC values (21). The existing studies regarding machine learning-based DWI prediction have preliminarily demonstrated the feasibility of predicting HER2 status from DWI, achieving the moderate performance about AUC of 0.80 level (7–12). These preliminary results revealed the inherent physiological link between DWI and HER2 status. Furthermore, we exploited DL to predict HER2 status from DWI and achieved a high predictive performance, which further reinforces the underlying physiological connection between DWI and HER2. Our work demonstrates the feasibility of only using DWI and DL to predict HER2 status of breast cancer in clinical applications in the future.

The proposed method achieved a similar classification performance compared to that reported in previous DCE-MRI studies (AUC of 0.71-0.90), which applied DL in DCE-MRI for HER2 status classification (15–20). DCE-MRI dynamically monitors the blood perfusion to quantify tissue microvascular function and pathophysiology, thereby establishing a physiological basis for HER2 classification. Nevertheless, the clinical application of DCE-MRI has certain limitations: it requires contrast agent injection and a longer scan time, which may introduce additional risks, and some patients cannot undergo the examination due to allergies or an inability to tolerate the lengthy scan. Therefore, when DCE-MRI is not feasible, DWI—a technique that requires no contrast agent, is simple to perform, and also is fast—can serve as an effective alternative.

The extensive research (33) in DL-based medical image classification has demonstrated that DL can more effectively extract the latent image features than clinical experts, leading to superior performance. Consistent with this, our proposed DL model significantly outperformed the clinical experts (0.91 vs. 0.65). This aligns with the known difficulty experts face in visually identifying lesion characteristics in breast DWI images (34). Furthermore, the DL model’s attention focused on the lesion region, as evidenced by the heatmap analysis (**Figure 5**), validates the rationale of the DL approach. These results underscore the strong potential of integrating MRI with DL for HER2 status prediction in breast cancer.

Our study has some limitations. First, the sample size, especially the external test set, is still relatively small, resulting in wide confidence intervals and limited statistical power for pairwise comparisons. Although this study provides useful insights into DWI-based HER2 status classification, the performance estimates should be interpreted as preliminary. In the future, the larger multi-center prospective validation is needed. Second, constrained by the availability of clinical data, our research was not designed for a three-category HER2 classification (zero, low, high expression). Instead, the primary objective was to reveal the feasibility of HER2 classification using only DWI images. Our subsequent research will explore the implementation of three-category classification with breast DWI.

In conclusion, this study suggests the feasibility of a hybrid CNN-Transformer model for non-invasive HER2 status classification from breast DWI, with encouraging discriminative performance in both internal and external test sets. Given the limited sample size and the retrospective design, these findings should be considered preliminary, and larger prospective multi-center studies are warranted before clinical translation.

## Data Availability

The raw data supporting the conclusions of this article will be made available by the authors, without undue reservation.
