# Supplementary material for: Deep learning-based prediction of HER2 status from breast diffusion-weighted MRI
Source: Front Oncol. 2026 Oct 5;16:1863720. doi: 10.3389/fonc.2026.1863720 (PMC13644321; doi:10.3389/fonc.2026.1863720)
Supplement: Supplementary file 1 [file DataSheet1.docx]

***Supplementary Material***

This Supplementary Material provides:

1) The details of the model architecture (**Supplementary Figure 1 and Table 1**);

2) The paired differences between our model and benchmark models using pre-specified fixed threshold of 0.5 (**Supplementary Table 2-Table 4**);

3) The paired differences between our model and benchmark models using model-specific Youden index threshold (**Supplementary Table 5-Table 9 and Figure 2**);

4) The paired differences between our model and clinical readers (**Supplementary Table 10 and Table 11**);

5) Training Loss Curve of the Proposed Model (**Supplementary Figure 3**).

**1. Model Architecture**

Supplementary Figure 1 shows the proposed model, which comprises three core modules: adaptive hybrid pooling (AHP), feature fusion CNN (FF_CNN), and 3D channel attention Transformer (3DCA_Trans). First, the input 3D DWI images are passed through a 3D convolutional layer. Subsequently, the features are processed and down-sampled by four stacked "AHP + FF_CNN" blocks. Finally, the output features are input to the 3DCA_Trans module, which produces the classification result.

**Supplementary Figure 1.** Architecture for HER2 detection with the proposed hybrid CNN-Transformer model. The model integrates three core modules: a feature fusion convolutional neural network (FF_CNN) module, an adaptive hybrid pooling (AHP) module, and a 3D channel attention transformer (3DCA_Trans) module.

**1.1 AHP**

Supplementary Figure 1A illustrates the AHP module [1], which receives a feature map as input and outputs a spatially down-sampled feature map. Its implementation involves a weighted fusion of max-pooling and average-pooling branches.

First, the module directs the input feature map into two parallel branches: one branch incorporates a "GN + GELU activation function + Conv3d + max pooling" sequence, while the other branch encompasses a "GN + GELU activation function + Conv3d + average pooling" sequence. Subsequently, the outputs of the two branches are weighted and fused with parameter α = 0.9 $\left( F_{fuse}=\alpha\cdot F_{avg}+\left（ 1-\alpha\right）\cdot F_{max} \right)$, thereby generating the fused pooling output.

**1.2 FF_CNN**

Supplementary Figure 1B illustrates the FF_CNN module, designed with inspiration from the DenseNet [2] architecture. This module takes an input feature map and outputs a feature map with an increased channel count, achieving feature fusion through a series of convolutional and feature concatenation operations.

First, the FF_CNN module applies two consecutive "group normalization (GN) + 3D convolution (Conv3d)" operations to the input feature map. Subsequently, the output of the second 3D convolutional layer is channel-concatenated with the module's initial input feature map. This process increases the model's channel count (each FF_CNN block adds 12 channels), ultimately yielding a feature map with an augmented channel count (12 × number of FFNs).

**1.3 3DCA_Trans**

Supplementary Figure 1C illustrates the 3DCA_Trans module [3], which receives a 4D feature map as input and transforms it into a series of channel embeddings. These are then processed by a Transformer architecture to output features for final classification.

First, the module flattens the input [Batch, C, D, H, W] feature map into [Batch, C, D × H × W]. Subsequently, a linear projection converts this into the serialized tokens [Batch, C, 128] required by the Transformer encoder. In this representation, C is treated as the sequence length, with each channel serving as a "token". These tokens are then fed into the Transformer encoder for the self-attention mechanism computation. Finally, the output of the Transformer encoder is passed through a multi-layer perceptron (MLP Head) for classification, ultimately yielding the classification result (HER2- or HER2+).

**1.4 Hybrid CNN-Transformer Architecture**

Supplementary Table 1 summarizes the input and output dimensions, key operations, and parameters of all model modules.

**Supplementary Table 1.** The architecture of the proposed 3D hybrid CNN-Transformer model

| Module  (stage) | Input Dimensions  (C × W × H × D) | Key Operations  or Parameters | Output Dimensions  (C × W × H × D) |
| --- | --- | --- | --- |
| Input Data | 3 × 128 × 128 × 32 | 3D DWI images | 3 × 128 × 128 × 32 |
| Conv3d | 3 × 128 × 128 × 32 | 3D Conv (7 × 7 × 7, 2, 3) | 64 × 64 × 64 × 16 |
| AHP (1) | 64 × 64 × 64 × 16 | No 3D Conv  3D Pool (3 × 3 × 3, 2, 1) | 64 × 32 × 32 × 8 |
| FF_CNN (1) | 64 × 32 × 32 × 8 | 6 FF_CNN  (adds 72 channels)  3D Conv (1 × 1 × 1, 1, 0)  3D Conv (3 × 3 × 3, 1, 1) | 136 × 32 × 32 × 8 |
| AHP (2) | 136 × 32 × 32 × 8 | 3D Conv (1 × 1 × 1, 1, 0)  3D Pool (2 × 2 × 2, 2, 0) | 68 × 16 × 16 × 4 |
| FF_CNN (2) | 68 × 16 × 16 × 4 | 12 FF_CNN  (adds 144 channels)  3D Conv (1 × 1 × 1, 1, 0)  3D Conv (3 × 3 × 3, 1, 1) | 212 × 16 × 16 × 4 |
| AHP (3) | 212 × 16 × 16 × 4 | 3D Conv (1 × 1 × 1, 1, 0)  3D Pool (2 × 2 × 2, 2, 0) | 106 × 8 × 8 × 4 |
| FF_CNN (3) | 106 × 8 × 8 × 4 | 24 FF_CNN  (adds 288 channels)  3D Conv (1 × 1 × 1, 1, 0)  3D Conv (3 × 3 × 3, 1, 1) | 394 × 8 × 8 × 4 |
| AHP (4) | 394 × 8 × 8 × 4 | 3D Conv (1 × 1 × 1, 1, 0)  3D Pool (2 × 2 × 2, 2, 0) | 197 × 4 × 4 × 1 |
| FF_CNN (4) | 197 × 4 × 4 × 1 | 16 FF_CNN  (adds 192 channels)  3D Conv (1 × 1 × 1, 1, 0)  3D Conv (3 × 3 × 3, 1, 1) | 389 × 4 × 4 × 1 |
| 3DCA_Trans | 389 × 4 × 4 × 1 | Transformer Module | 2 |

In the key operations or parameters column, parentheses indicate (kernel size, stride, padding).

**2. Paired Differences** **between Our Model and Benchmark Models (using pre-specified fixed threshold of 0.5)**

**Supplementary Table 2.** Paired comparisons between our model and benchmark models in the internal test set

| **Paired comparison** | **Metric** | **Paired difference, Δ (95% CI)** | **P value** |
| --- | --- | --- | --- |
| Our Model vs ViT | AUC | 0.106 (−0.051, 0.263) | 0.186 |
| Our Model vs ViT | Accuracy | 0.103 (−0.062, 0.265) | 0.206 |
| Our Model vs ViT | Sensitivity | −0.056 (−0.289, 0.179) | 0.564 |
| Our Model vs ViT | Specificity | 0.238 (−0.010, 0.461) | 0.059 |
| Our Model vs ViT | F1 score | 0.082 (−0.077, 0.253) | 0.318 |
| Our Model vs DenseNet | AUC | 0.095 (−0.040, 0.230) | 0.166 |
| Our Model vs DenseNet | Accuracy | 0.128 (−0.031, 0.286) | 0.096 |
| Our Model vs DenseNet | Sensitivity | 0.056 (−0.206, 0.310) | 0.655 |
| Our Model vs DenseNet | Specificity | 0.190 (−0.017, 0.402) | **0.046** |
| Our Model vs DenseNet | F1 score | 0.126 (−0.041, 0.312) | 0.158 |
| Our Model vs ResNet | AUC | 0.077 (−0.049, 0.203) | 0.233 |
| Our Model vs ResNet | Accuracy | 0.051 (−0.090, 0.195) | 0.414 |
| Our Model vs ResNet | Sensitivity | 0.000 (−0.250, 0.250) | 1.000 |
| Our Model vs ResNet | Specificity | 0.095 (−0.087, 0.294) | 0.157 |
| Our Model vs ResNet | F1 score | 0.049 (−0.099, 0.207) | 0.532 |
| Our Model vs ConvNeXt | AUC | 0.040 (−0.068, 0.148) | 0.471 |
| Our Model vs ConvNeXt | Accuracy | 0.051 (−0.090, 0.195) | 0.414 |
| Our Model vs ConvNeXt | Sensitivity | −0.056 (−0.289, 0.179) | 0.564 |
| Our Model vs ConvNeXt | Specificity | 0.143 (−0.052, 0.349) | 0.083 |
| Our Model vs ConvNeXt | F1 score | 0.040 (−0.098, 0.187) | 0.570 |

Data are presented as point estimates and paired differences with 95% confidence intervals. Binary classification metrics were calculated using the fixed 0.5 decision threshold. Differences are calculated as Our Model minus the benchmark.

**Supplementary Table 3.** Paired comparisons between our model and benchmark models in the external test set

| **Paired comparison** | **Metric** | **Paired difference, Δ (95% CI)** | **P value** |
| --- | --- | --- | --- |
| Our Model vs ViT | AUC | 0.096 (−0.050, 0.242) | 0.196 |
| Our Model vs ViT | Accuracy | 0.023 (−0.141, 0.185) | 0.782 |
| Our Model vs ViT | Sensitivity | 0.045 (−0.172, 0.261) | 0.655 |
| Our Model vs ViT | Specificity | 0.000 (−0.246, 0.246) | 1.000 |
| Our Model vs ViT | F1 score | 0.025 (−0.127, 0.179) | 0.754 |
| Our Model vs DenseNet | AUC | 0.084 (−0.068, 0.235) | 0.279 |
| Our Model vs DenseNet | Accuracy | 0.182 (0.014, 0.339) | **0.033** |
| Our Model vs DenseNet | Sensitivity | 0.136 (−0.107, 0.364) | 0.257 |
| Our Model vs DenseNet | Specificity | 0.227 (−0.011, 0.432) | 0.059 |
| Our Model vs DenseNet | F1 score | 0.157 (0.008, 0.323) | 0.051 |
| Our Model vs ResNet | AUC | 0.070 (−0.082, 0.222) | 0.365 |
| Our Model vs ResNet | Accuracy | 0.159 (−0.034, 0.338) | 0.108 |
| Our Model vs ResNet | Sensitivity | 0.500 (0.215, 0.692) | **0.002** |
| Our Model vs ResNet | Specificity | −0.182 (−0.397, 0.044) | 0.102 |
| Our Model vs ResNet | F1 score | 0.289 (0.057, 0.542) | **0.021** |
| Our Model vs ConvNeXt | AUC | 0.041 (−0.086, 0.168) | 0.524 |
| Our Model vs ConvNeXt | Accuracy | 0.045 (−0.077, 0.170) | 0.414 |
| Our Model vs ConvNeXt | Sensitivity | 0.136 (−0.043, 0.327) | 0.083 |
| Our Model vs ConvNeXt | Specificity | −0.045 (−0.231, 0.140) | 0.564 |
| Our Model vs ConvNeXt | F1 score | 0.060 (−0.042, 0.181) | 0.277 |

Data are presented as point estimates and paired differences with 95% confidence intervals. Binary classification metrics were calculated using the fixed 0.5 decision threshold. Differences are calculated as Our Model minus the benchmark.

**Supplementary Table 4.** Paired comparisons between our model and benchmark models in the combined internal and external test sets (n = 83)

| **Paired comparison** | **Metric** | **Paired difference, Δ (95% CI)** | **P value** |
| --- | --- | --- | --- |
| Our Model vs ViT | AUC | 0.094 (−0.015, 0.204) | 0.091 |
| Our Model vs ViT | Accuracy | 0.060 (−0.054, 0.174) | 0.297 |
| Our Model vs ViT | Sensitivity | 0.000 (−0.153, 0.153) | 1.000 |
| Our Model vs ViT | Specificity | 0.116 (−0.061, 0.286) | 0.197 |
| Our Model vs ViT | F1 score | 0.050 (−0.058, 0.162) | 0.369 |
| Our Model vs DenseNet | AUC | 0.105 (0.008, 0.202) | **0.033** |
| Our Model vs DenseNet | Accuracy | 0.157 (0.044, 0.267) | **0.007** |
| Our Model vs DenseNet | Sensitivity | 0.100 (−0.072, 0.267) | 0.248 |
| Our Model vs DenseNet | Specificity | 0.209 (0.061, 0.352) | **0.007** |
| Our Model vs DenseNet | F1 score | 0.143 (0.032, 0.264) | **0.017** |
| Our Model vs ResNet | AUC | 0.097 (−0.020, 0.214) | 0.104 |
| Our Model vs ResNet | Accuracy | 0.108 (−0.011, 0.225) | 0.072 |
| Our Model vs ResNet | Sensitivity | 0.275 (0.082, 0.444) | **0.008** |
| Our Model vs ResNet | Specificity | −0.047 (−0.191, 0.097) | 0.480 |
| Our Model vs ResNet | F1 score | 0.158 (0.019, 0.308) | **0.033** |
| Our Model vs ConvNeXt | AUC | 0.047 (−0.036, 0.129) | 0.267 |
| Our Model vs ConvNeXt | Accuracy | 0.048 (−0.039, 0.137) | 0.248 |
| Our Model vs ConvNeXt | Sensitivity | 0.050 (−0.086, 0.189) | 0.414 |
| Our Model vs ConvNeXt | Specificity | 0.047 (−0.079, 0.175) | 0.414 |
| Our Model vs ConvNeXt | F1 score | 0.049 (−0.034, 0.139) | 0.263 |

Data are presented as point estimates and paired differences with 95% confidence intervals. Binary classification metrics were calculated using the fixed 0.5 decision threshold. Differences are calculated as Our Model minus the benchmark.

**3. Paired Differences between Our Model and Benchmark Models (using model-specific Youden index threshold).**

**Supplementary Table 5.** Performance comparison between the proposed hybrid CNN-Transformer model and benchmark models in the internal test set

| Method | AUC | Accuracy | Sensitivity | Specificity | F1 score |
| --- | --- | --- | --- | --- | --- |
| ViT | 0.82 (0.69, 0.92) | 0.82 (0.69, 0.92) | 0.89 (0.73, 1.00) | 0.76 (0.57, 0.94) | 0.82 (0.65, 0.93) |
| DenseNet | 0.83 (0.68, 0.95) | 0.82 (0.69, 0.92) | 0.78 (0.57, 0.95) | 0.86 (0.70, 1.00) | 0.80 (0.62, 0.93) |
| ResNet | 0.85 (0.72, 0.96) | 0.85 (0.72, 0.92) | 0.83 (0.64, 1.00) | 0.86 (0.70, 1.00) | 0.83 (0.67, 0.95) |
| ConvNeXt | 0.89 (0.76, 0.98) | 0.85 (0.72, 0.95) | **0.89 (0.73, 1.00)** | 0.81 (0.63, 0.95) | 0.84 (0.69, 0.95) |
| **Our Model** | **0.93 (0.82, 0.99)** | **0.90 (0.80, 0.97)** | 0.83 (0.65, 1.00) | **0.95 (0.84, 1.00)** | **0.88 (0.75, 0.97)** |

The best results are shown in bold. The values in parentheses are confidence intervals. AUC, area under the receiver operating characteristic curve. Binary classification metrics were calculated using model-specific Youden index threshold

**Supplementary Table 6.** Performance comparison between the proposed hybrid CNN-Transformer model and benchmark models in the external test set

| Method | AUC | Accuracy | Sensitivity | Specificity | F1 score |
| --- | --- | --- | --- | --- | --- |
| ViT | 0.81 (0.67, 0.94) | 0.82 (0.70, 0.93) | 0.86 (0.70, 1.00) | 0.77 (0.59, 0.95) | 0.81 (0.67, 0.92) |
| DenseNet | 0.82 (0.68, 0.94) | 0.82 (0.70, 0.93) | 0.77 (0.60, 0.95) | 0.86 (0.71, 1.00) | 0.81 (0.67, 0.92) |
| ResNet | 0.84 (0.68, 0.96) | 0.82 (0.70, 0.93) | 0.82 (0.62, 0.96) | 0.82 (0.64, 0.96) | 0.82 (0.68, 0.93) |
| ConvNeXt | 0.87 (0.73, 0.97) | 0.84 (0.73, 0.93) | **0.91 (0.77, 1.00)** | 0.77 (0.59, 0.95) | 0.85 (0.72, 0.94) |
| **Our Model** | **0.91 (0.79, 0.99)** | **0.89 (0.77, 0.97)** | 0.86 (0.70, 1.00) | **0.91 (0.76, 1.00)** | **0.88 (0.75, 0.97)** |

The best results are shown in bold. The values in parentheses are confidence intervals. AUC, area under the receiver operating characteristic curve. Binary classification metrics were calculated using model-specific Youden index threshold

**Supplementary Table 7.** Paired comparisons between our model and benchmark models in the internal test set

| **Paired comparison** | **Metric** | **Paired difference, Δ (95% CI)** | **P value** |
| --- | --- | --- | --- |
| Our Model vs ViT | AUC | 0.106 (−0.051, 0.263) | 0.186 |
| Our Model vs ViT | Accuracy | 0.077 (−0.084, 0.237) | 0.317 |
| Our Model vs ViT | Sensitivity | −0.056 (−0.289, 0.179) | 0.564 |
| Our Model vs ViT | Specificity | 0.190 (−0.045, 0.412) | 0.102 |
| Our Model vs ViT | F1 score | 0.062 (−0.095, 0.227) | 0.445 |
| Our Model vs DenseNet | AUC | 0.095 (−0.040, 0.230) | 0.166 |
| Our Model vs DenseNet | Accuracy | 0.077 (−0.070, 0.226) | 0.257 |
| Our Model vs DenseNet | Sensitivity | 0.056 (−0.206, 0.310) | 0.655 |
| Our Model vs DenseNet | Specificity | 0.095 (−0.087, 0.294) | 0.157 |
| Our Model vs DenseNet | F1 score | 0.082 (−0.078, 0.260) | 0.337 |
| Our Model vs ResNet | AUC | 0.077 (−0.049, 0.203) | 0.233 |
| Our Model vs ResNet | Accuracy | 0.051 (−0.090, 0.195) | 0.414 |
| Our Model vs ResNet | Sensitivity | 0.000 (−0.250, 0.250) | 1.000 |
| Our Model vs ResNet | Specificity | 0.095 (−0.087, 0.294) | 0.157 |
| Our Model vs ResNet | F1 score | 0.049 (−0.099, 0.207) | 0.532 |
| Our Model vs ConvNeXt | AUC | 0.040 (−0.068, 0.148) | 0.471 |
| Our Model vs ConvNeXt | Accuracy | 0.051 (−0.090, 0.195) | 0.414 |
| Our Model vs ConvNeXt | Sensitivity | −0.056 (−0.289, 0.179) | 0.564 |
| Our Model vs ConvNeXt | Specificity | 0.143 (−0.052, 0.349) | 0.083 |
| Our Model vs ConvNeXt | F1 score | 0.040 (−0.098, 0.187) | 0.570 |

Data are presented as point estimates and paired differences with 95% confidence intervals. Binary classification metrics were calculated using model-specific Youden index threshold. Differences are calculated as Our Model minus the benchmark.

**Supplementary Table 8.** Paired comparisons between our model and benchmark models in the external test set

| **Paired comparison** | **Metric** | **Paired difference, Δ (95% CI)** | **P value** |
| --- | --- | --- | --- |
| Our Model vs ViT | AUC | 0.096 (−0.050, 0.242) | 0.196 |
| Our Model vs ViT | Accuracy | 0.068 (−0.077, 0.214) | 0.317 |
| Our Model vs ViT | Sensitivity | 0.000 (−0.213, 0.213) | 1.000 |
| Our Model vs ViT | Specificity | 0.136 (−0.088, 0.353) | 0.180 |
| Our Model vs ViT | F1 score | 0.058 (−0.073, 0.198) | 0.400 |
| Our Model vs DenseNet | AUC | 0.084 (−0.068, 0.235) | 0.279 |
| Our Model vs DenseNet | Accuracy | 0.068 (−0.097, 0.230) | 0.405 |
| Our Model vs DenseNet | Sensitivity | 0.091 (−0.165, 0.333) | 0.480 |
| Our Model vs DenseNet | Specificity | 0.045 (−0.172, 0.261) | 0.655 |
| Our Model vs DenseNet | F1 score | 0.074 (−0.100, 0.259) | 0.414 |
| Our Model vs ResNet | AUC | 0.070 (−0.082, 0.222) | 0.365 |
| Our Model vs ResNet | Accuracy | 0.068 (−0.077, 0.214) | 0.317 |
| Our Model vs ResNet | Sensitivity | 0.045 (−0.180, 0.267) | 0.655 |
| Our Model vs ResNet | Specificity | 0.091 (−0.120, 0.301) | 0.317 |
| Our Model vs ResNet | F1 score | 0.066 (−0.072, 0.216) | 0.367 |
| Our Model vs ConvNeXt | AUC | 0.041 (−0.086, 0.168) | 0.524 |
| Our Model vs ConvNeXt | Accuracy | 0.045 (−0.079, 0.174) | 0.414 |
| Our Model vs ConvNeXt | Sensitivity | −0.045 (−0.246, 0.153) | 0.564 |
| Our Model vs ConvNeXt | Specificity | 0.136 (−0.043, 0.327) | 0.083 |
| Our Model vs ConvNeXt | F1 score | 0.033 (−0.076, 0.147) | 0.564 |

Data are presented as point estimates and paired differences with 95% confidence intervals. Binary classification metrics were calculated using model-specific Youden index threshold. Differences are calculated as Our Model minus the benchmark.

**Supplementary Table 9.** Paired comparisons between our model and benchmark models in the combined internal and external test sets (n = 83)

| **Paired comparison** | **Metric** | **Paired difference, Δ (95% CI)** | **P value** |
| --- | --- | --- | --- |
| Our Model vs ViT | AUC | 0.094 (−0.015, 0.204) | 0.091 |
| Our Model vs ViT | Accuracy | 0.072 (−0.032, 0.177) | 0.157 |
| Our Model vs ViT | Sensitivity | −0.025 (−0.172, 0.121) | 0.705 |
| Our Model vs ViT | Specificity | 0.163 (0.009, 0.315) | **0.035** |
| Our Model vs ViT | F1 score | 0.060 (−0.041, 0.164) | 0.248 |
| Our Model vs DenseNet | AUC | 0.105 (0.008, 0.202) | **0.033** |
| Our Model vs DenseNet | Accuracy | 0.072 (−0.037, 0.181) | 0.180 |
| Our Model vs DenseNet | Sensitivity | 0.075 (−0.103, 0.249) | 0.405 |
| Our Model vs DenseNet | Specificity | 0.070 (−0.067, 0.210) | 0.257 |
| Our Model vs DenseNet | F1 score | 0.078 (−0.042, 0.203) | 0.210 |
| Our Model vs ResNet | AUC | 0.097 (−0.020, 0.214) | 0.104 |
| Our Model vs ResNet | Accuracy | 0.060 (−0.036, 0.158) | 0.197 |
| Our Model vs ResNet | Sensitivity | 0.025 (−0.134, 0.184) | 0.739 |
| Our Model vs ResNet | Specificity | 0.093 (−0.031, 0.226) | 0.102 |
| Our Model vs ResNet | F1 score | 0.058 (−0.042, 0.163) | 0.260 |
| Our Model vs ConvNeXt | AUC | 0.047 (−0.036, 0.129) | 0.267 |
| Our Model vs ConvNeXt | Accuracy | 0.048 (−0.039, 0.138) | 0.248 |
| Our Model vs ConvNeXt | Sensitivity | −0.050 (−0.191, 0.088) | 0.414 |
| Our Model vs ConvNeXt | Specificity | 0.140 (0.024, 0.270) | **0.014** |
| Our Model vs ConvNeXt | F1 score | 0.036 (−0.049, 0.124) | 0.404 |

Data are presented as point estimates and paired differences with 95% confidence intervals. Binary classification metrics were calculated using model-specific Youden index threshold. Differences are calculated as Our Model minus the benchmark.

**Supplementary Figure 2.** Confusion matrices for HER2 status classification by the proposed hybrid CNN-Transformer model in (A) the internal test set and (B) the external test set. Binary classification metrics were calculated using model-specific Youden index threshold.

**4. Paired Differences** **between Our Model and** **Clinical Readers**

**Supplementary Table 10.** Paired comparisons between our model and clinical readers in the external test set (The deep learning models derived binary classifications using pre-specified fixed threshold of 0.5)

| **Paired comparison** | **Metric** | **Paired difference, Δ (95% CI)** | **P value** |
| --- | --- | --- | --- |
| Our Model vs Reader 1 | AUC | 0.280 (0.089, 0.471) | **0.004** |
| Our Model vs Reader 1 | Accuracy | 0.273 (0.088, 0.435) | **0.005** |
| Our Model vs Reader 1 | Sensitivity | 0.273 (−0.006, 0.504) | 0.058 |
| Our Model vs Reader 1 | Specificity | 0.273 (0.022, 0.479) | **0.034** |
| Our Model vs Reader 1 | F1 score | 0.255 (0.082, 0.447) | **0.007** |
| Our Model vs Reader 2 | AUC | 0.261 (0.065, 0.457) | **0.009** |
| Our Model vs Reader 2 | Accuracy | 0.227 (0.041, 0.395) | **0.018** |
| Our Model vs Reader 2 | Sensitivity | 0.318 (0.029, 0.548) | **0.035** |
| Our Model vs Reader 2 | Specificity | 0.136 (−0.109, 0.361) | 0.257 |
| Our Model vs Reader 2 | F1 score | 0.246 (0.060, 0.453) | **0.016** |

Data are presented as point estimates and paired differences with 95% confidence intervals. Differences are calculated as Our Model minus the Reader.

**Supplementary Table 11.** Paired comparisons between our model and clinical readers in the external test set (The deep learning models derived binary classifications using model-specific Youden index threshold)

| **Paired comparison** | **Metric** | **Paired difference, Δ (95% CI)** | **P value** |
| --- | --- | --- | --- |
| Our Model vs Reader 1 | AUC | 0.280 (0.089, 0.471) | **0.004** |
| Our Model vs Reader 1 | Accuracy | 0.318 (0.131, 0.479) | **0.002** |
| Our Model vs Reader 1 | Sensitivity | 0.227 (−0.034, 0.454) | 0.096 |
| Our Model vs Reader 1 | Specificity | 0.409 (0.140, 0.613) | **0.007** |
| Our Model vs Reader 1 | F1 score | 0.288 (0.108, 0.487) | **0.004** |
| Our Model vs Reader 2 | AUC | 0.261 (0.065, 0.457) | **0.009** |
| Our Model vs Reader 2 | Accuracy | 0.273 (0.092, 0.433) | **0.005** |
| Our Model vs Reader 2 | Sensitivity | 0.273 (0.000, 0.500) | 0.058 |
| Our Model vs Reader 2 | Specificity | 0.273 (0.022, 0.490) | **0.034** |
| Our Model vs Reader 2 | F1 score | 0.279 (0.093, 0.489) | **0.008** |

Data are presented as point estimates and paired differences with 95% confidence intervals. Differences are calculated as Our Model minus the Reader.

**5. Training Loss Curve of the Proposed Model**

**Supplementary Figure 3.** Training loss curve of the proposed hybrid CNN-Transformer model on the 156-patient training set. The loss decreased rapidly and plateaued around 0.35 after approximately 60 epochs, indicating stable convergence; the plateau above zero is expected, possibly because online data augmentation continuously presents previously unseen sample variations in each epoch, and the residual oscillations reflect the small batch size (batch size = 2). Network parameters at the final epoch were used for all evaluations.

**References**

1.	Wang C, Cai Z, Zhang Y, Yang S. Physiology based unsupervised learning for segmentation of COVID-19 lesions in chest 3D CT scans. *Med Phys.* (2025) 52:e18049. doi: 10.1002/mp.18049

2.	Huang G, Liu Z, Van Der Maaten L, Weinberger KQ. Densely connected convolutional networks. In: *Proceedings of the IEEE conference on computer vision and pattern recognition.* Honolulu, HI, USA: IEEE (2017). p. 4700-8.

3.	Shi S, Wang C, Xiao S, Li H, Zhao X, Guo F, et al. Magnetic resonance image denoising for Rician noise using a novel hybrid transformer-CNN network (HTC-net) and self-supervised pretraining. *Med Phys.* (2025) 52:1643-60. doi: 10.1002/mp.17562
